# Domestic Correspondence

**Published:** 1890-04

**Authors:** 


### Domestic Correspondence.

To the Editor:

*Society "Incident."*—At a recent meeting of the New York Odontological Society, a gentleman present (not a member), in response to a general invitation by the president to relate "incidents of office practice," took advantage of the occasion for the purpose of reporting a case under his observation, which, in substance, was as follows:

"A lady was under his care for treatment. In her teeth were upward of forty fillings. Six of the teeth were devoid of vitality. Her teeth were also exceedingly loose,—a marked case of pyorrhœa alveolaris,—and both teeth and gums were causing much pain. Indeed, it was a bad case all around. The lady's former dentist was reported as a 'prominent' member of our specialty; prominent through his writings and teachings; a professor of operative dentistry in one of our 'prominent' dental colleges, located in one of our prominent cities, yet his name was modestly withheld.

"This prominent professor had permitted this lady's teeth to get into this deplorable condition, and the fact must be widely known and published, to the discredit and shame of this unworthy, yet 'prominent' professor, who, instead of occupying the professor's chair, might better take a seat on a bench with the 'boys' and learn how to do what he was pretending to teach to the youthful aspirants for diplomatic honors."

This prominent professor of the prominent college was certainly unworthy of his high calling,—at least was so pictured to the members of the Odontological Society, who patiently listened to the rehearsal of these woes. They could imagine this prominent professor as simply a fraud, a botch, a humbug; careless, remiss, incompetent, and totally unfit to practise dentistry. And yet he holds a position as a professor of operative dentistry in a prominent dental college! "Human impudence—to what a height hast thou arisen!" Why are such things permitted?

But, to be serious, why should such a case have been thus stated at a society meeting? What does it signify, except to traduce in a semi-blind manner a fellow-member of our calling? Who knows anything of the circumstances connected with the case referred to? Are dentists responsible for all the cavities that form in human teeth? Are they responsible for the neglect of individuals to have them timely attended to? Are they to be blamed because teeth are allowed to become incrusted with scales of salivary calculus and loaded with other filthy deposits? And are they to be blamed for all the dead teeth that exist? Is it not possible for an individual to have "six teeth with dead pulps" without being a victim of malpractice? Thousands upon thousands of dentures are covered with unsightly stains and their interstices filled with mischievous accumulations. Many mouths can make an exhibit of rows of pulpless teeth or crownless roots. Must the dentist, who, perhaps, has had but a semi-occasional opportunity afforded him by a careless patient to keep his or her teeth in order, and who visits him only when driven by desperate pain, or when mastication has become almost a "lost art," be blamed for not staying the inevitable results of carelessness or indifference?

Really, it is not a nice thing to do to bring up this sort of (so-called) "Incidents of office practice" before society gatherings. It is a decided breach of courtesy, and should be severely rebuked. Reports brought by patients should be taken with many grains of allowance. Few individuals are willing to blame themselves for their sins of omission, and especially is this the case if they can make a pack-horse of some unfortunate dentist on whom they can saddle the responsibility for the condition of their shocking bad dentures. Circumstances and conditions must be taken into account before judgment can be fairly passed. Who can get at the exact truth in such matters? One might as well try to correctly picture the life, character, habits, and disposition of a deceased stranger by viewing the corpse!

Be charitable, ye who dwell in glass houses, and let him only who is proof against criticism hurl the damaging missiles.

"X."

---

To the Editor:

A new organization has been formed among the dental students of the University of Pennsylvania under the name of the James Truman Dental Society. The first regular meeting was held on Thursday evening, March 13, 1890, in the college building. The following officers were elected for the ensuing year: J. A. McKee, Jr., president; Louis Stephan, vice-president; W. T. Arrington, Jr., secretary; George J. Frey, treasurer. The society offers to its members a field for discussion and active investigation of all such subjects and questions that may be suggested in their course of study.

W. T. Arrington Jr., *Secretary.*

